# Loop diuretics in acute heart failure: beyond the decongestive relief for the kidney

**DOI:** 10.1186/s13054-015-1017-3

**Published:** 2015-09-03

**Authors:** Alberto Palazzuoli, Gaetano Ruocco, Claudio Ronco, Peter A. McCullough

**Affiliations:** Department of Internal and Surgical Medicine, Cardiovascular Diseases Unit, Le Scotte Hospital, University of Siena Viale Bracci, 53100 Siena, Italy; Nephrology Dialysis & Transplantation International Renal Research Institute (IRRIV) St. Bortolo Hospital, Viale Rodolfi 37, IT-36100 Vicenza, Italy; Baylor University Medical Center, Baylor Heart and Vascular Institute, Baylor Jack and Jane Hamilton Heart and Vascular Hospital, 621 North Hall Street, H030, Dallas, Texas 75226 USA

## Abstract

Current goals in the acute treatment of heart failure are focused on pulmonary and systemic decongestion with loop diuretics as the cornerstone of therapy. Despite rapid relief of symptoms in patients with acute decompensated heart failure, after intravenous use of loop diuretics, the use of these agents has been consistently associated with adverse events, including hypokalemia, azotemia, hypotension, and increased mortality. Two recent randomized trials have shown that continuous infusions of loop diuretics did not offer benefit but were associated with adverse events, including hyponatremia, prolonged hospital stay, and increased rate of readmissions. This is probably due to the limitations of congestion evaluation as well as to the deleterious effects linked to drug administration, particularly at higher dosage. The impaired renal function often associated with this treatment is not extensively explored and could deserve more specific studies. Several questions remain to be answered about the best diuretic modality administration, global clinical impact during acute and post-discharge period, and the role of renal function deterioration during treatment. Thus, if loop diuretics are a necessary part of the treatment for acute heart failure, then there must be an approach that allows personalization of therapy for optimal benefit and avoidance of adverse events.

## Introduction

In patients with acute heart failure (AHF), the risk of death or rehospitalization within 60 days from admission ranges from 30 to 60 %, depending on the population studied [1, 2]. The symptoms that drive hospital admission are linked to congestion, and loop diuretics are the most common initial therapeutic approach (used in 90 % of cases) [3, 4]. These agents promptly improve symptoms and have been shown to reduce dyspnea scores and peripheral edema [4, 5]. However, loop diuretics have been associated with increased rates of mortality and readmission in a graded fashion with cumulative dose and with continuous infusions [6, 7]. It is unclear whether this association is due to confounding by indication or whether adverse events lead to clinically meaningful outcomes accounting for these observations. In this context, neither European Society of Cardiology nor American College of Cardiology/American Heart Association guidelines provide any specific recommendation regarding starting and maintaining dosage, oral or intravenous infusion, and time course of treatment [1]. Thus, despite the clinical efficacy of these drugs in providing decongestion and symptomatic improvement, several questions remain to be answered about the optimal approach in any given patient providing diuresis and decongestion but not tipping the balance leading to acute kidney injury (AKI), electrolyte disturbances, and worse outcomes.

## Congestion in heart failure

It has been difficult to demonstrate a uniform benefit with respect to any individual therapeutic intervention in patients with AHF [3–6]. This is probably due to a wide range of pathophysiologies that result in a common phenotypic appearance of pulmonary congestion and peripheral edema [8, 9]. Although pulmonary and systemic congestion may be the most overt findings, these may be the “tip of the iceberg” reflecting significant congestion of multiple organs, including the kidneys. Many cardiac and extracardiac factors can lead to congestion: left ventricular (LV) adverse remodeling, hypertrophy, and stiffness; coronary artery disease and microvascular ischemia; decreased systemic vascular compliance; reduced venous capacitance and excessive preload; superimposed right ventricular dysfunction; and pulmonary hypertension. Neurohormonal determinants include renin-angiotensin activation, nervous sympathetic overdrive, increased arginine-vasopressin activity, endothelin secretion, and increased immune cell signaling. Clinically, the patient with AHF can be categorized into a two-by-two table according to good or poor systemic perfusion and to the presence or absence of congestion [10]. Those with both poor perfusion and congestion have the worst overall risk for short- and long-term mortality as well as worsened renal function after the initiation of intravenous loop diuretics.

Beyond hemodynamic derangement, there are systemic mechanisms that substantially contribute to congestion status, including neurohormonal activation which works to maintain organ perfusion and systemic pressure while enhancing sodium and water retention. Combined, increased vascular stiffness and reduced vein capacitance enhance both preload and afterload with a consequent rise in cardiac filling pressure that reflects on the pulmonary circulation. Arterial vasoconstriction associated with increased venous pressure affects the kidney, causing hemodynamic and parenchymal alterations: renal blood flow redistribution, tubuloglomerular feedback, tubule obliteration, and efferent arteriolar constriction which leads to reduced salt and water excretion. Slow plasma refill from extravascular to intravascular bed and overhydrated interstitium is another component: the intravascular volume decreases only marginally by diuretic treatment; in contrast, the interstitial volume has substantial reduction.

**Fig. 1:**
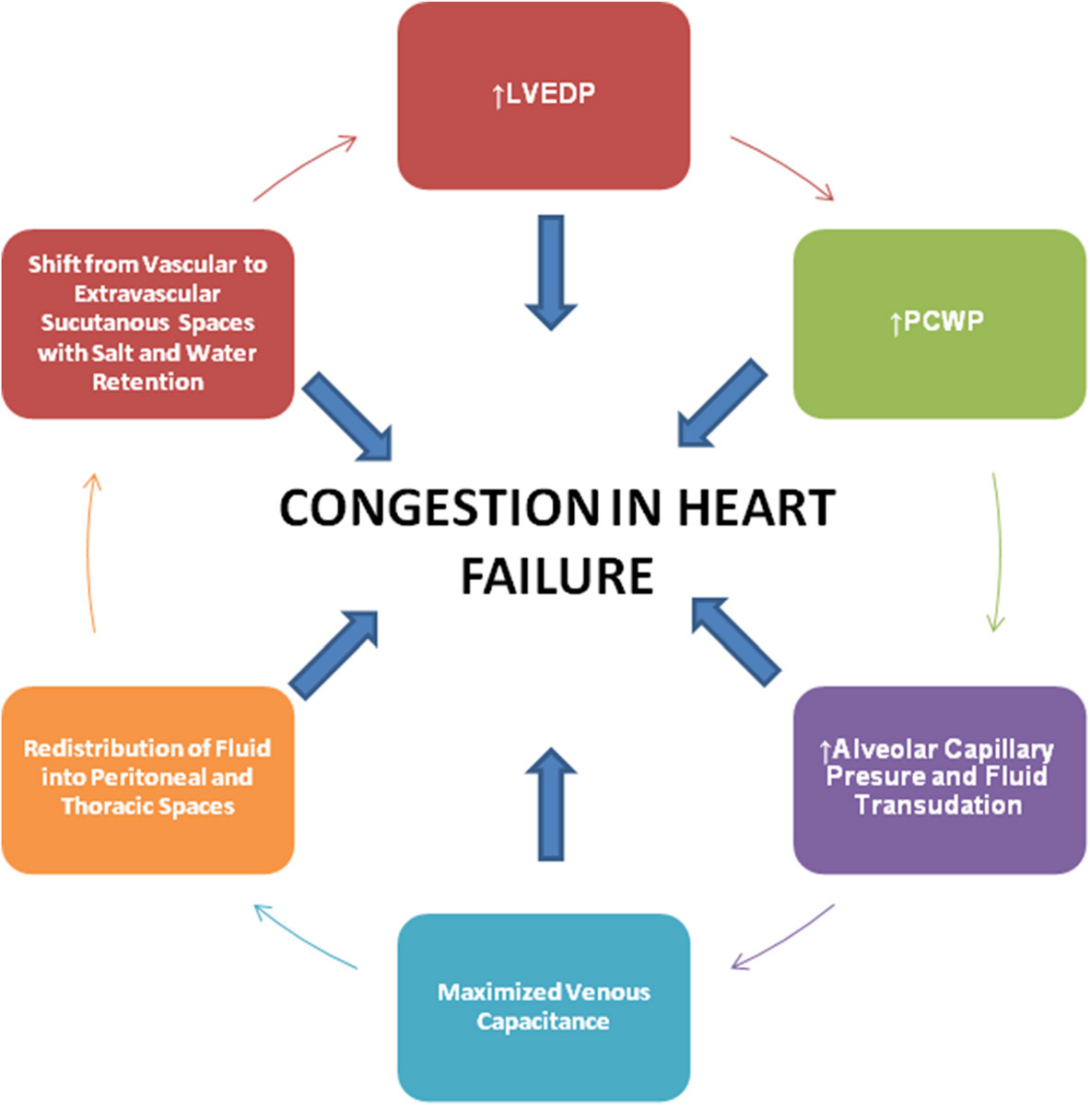
Potential factors contributing to the development of congestion in acute heart failure. *LVEDP* left ventricular end-diastolic pressure, *PCWP* pulmonary capillary wedge pressure

Given the combination of hemodynamic and neurohormonal factors, each patient has an individual risk of AKI or type 1 cardiorenal syndrome after the first few doses of intravenous loop diuretics [11]. A strong clinical clue for risk of AKI is increased central venous pressure and therefore intra-abdominal and renal congestion. Elevated intra-abdominal pressure has recently been debated as a causal contributor in congestion development. Raised intra-abdominal pressure reduces abdominal compliance, leading to intra-abdominal organ compression due to reduced venous return and parenchymal congestion [12]. Moreover, elevated intra-abdominal pressure may raise central venous pressure, which consequently increases cardiac preload (Fig. 1). If the degree of diuresis exceeds the rate of plasma refill from the extravascular space, then transient decreases in forward flow to the kidney can occur with the same amount of venous back pressure, precipitating tubuloglomerular feedback and a reduction of glomerular filtration combined with augmented sodium reabsorption. This causes an abrupt reduction in urine output and then a rise in the plasma pool of creatinine, urea, and other filtered substances.

The physical exam and traditional laboratories are poor tools in assessing this complex and dynamic pathophysiology in each patient. Futuristic approaches involve the use of non-invasive abdominal compartment monitoring, bioimpedance, or real-time assessments of glomerular filtration to establish the correct loop diuretic dosing and management of other drugs.

## Significance of worsening renal function during treatment

The rates of AKI or type 1 cardiorenal syndrome range from 20 to 70 %, depending on different definitions, creatinine cutpoint values, and the study population [13–15]. Loop diuretic administration, particularly higher doses and continuous infusions, is associated with higher rates of AKI. Loop diuretics markedly increase activation of the renin-angiotensin and sympathetic nervous systems. This phenomenon is mediated by two distinct mechanisms: the inhibition of sodium chloride uptake into the macula densa cells and the stimulation of prostacyclin that further increases secretion of renin. Indirect effects of loop diuretics include reductions of renal blood flow and enhanced proximal tubule sodium reabsorption in between loop diuretic doses. Chronically, these cells hypertrophy and counteract the effect of loop diuretics. Finally, aldosterone works at the collecting duct to recover remaining sodium in the urine while increased arginine vasopressin can recover as much as 25 % of the free water in urine despite the use of loop diuretics. Elevated diuretic dosages are associated with increased sympathetic overdrive mediated by direct and indirect renin activity. Indirect effects of diuretic are observed in several sites: at glomerulus by reduction of renal blood flow, at proximal tubules by increase of sodium resorption, and at collection duct by aldosterone activity. All of these modifications lead to delivery of sodium to the distal nephron, increased sodium reabsorption, and distal tubule hypertrophy. The above functional and parenchymal kidney modifications could potentially result in sudden creatinine increase and epidermal growth factor receptor reduction, amplifying the AKI independently from primary kidney disease [16]. Two recent reports have shown that impaired renal function after discharge had a worse prognosis compared with in-hospital AKI; the same reports suggested that, in the post-discharge period, diuretic resistance can persist and that renal function should also be monitored closely [17, 18]. The importance of renal function changes during hospitalization and its relation to decongestion has been confirmed by a retrospective analysis of the ESCAPE trial (Evaluation Study of Congestive Heart Failure and Pulmonary Artery Catheterization Effectiveness Trial) in which hemoconcentration as a marker of more intense decongestion was associated with a better prognosis even if increased blood urea nitrogen (BUN) was observed [19]. Hence, there is a great need for methods to discern between transient changes in serum creatinine likely associated with overdiuresis on one hand and AKI with more long-lasting and injurious insults to the kidney on the other [20]. However, data from the Diuretic Optimization Strategies Evaluation (DOSE) AHF Trial demonstrated that patients randomly assigned to higher doses of loop diuretics had higher rates of AKI but similar outcomes with respect to rehospitalization and death [21]. Additionally, in a recent prospective study including two groups of patients receiving a high diuretic dosage in different administrations (intermittent versus continuous), we found that more active diuresis and weight loss were associated with higher rates of AKI, increased risk of death, and readmission at 6 months [22]. When fluid removal exceeds the rate of plasma refill in those undergoing ultrafiltration, AKI can be observed in a similar fashion to that with loop diuretics [23]. Other attempts to obtain effective decongestion that reduces diuretic dosage by using other drugs have recently failed: neither low-dose dopamine nor low-dose nesiritide enhanced decongestion or improved renal function [24]. These recent findings suggest that the relation between decongestion, transient or persistent AKI, and diuretic management is poorly understood. Transient AKI could be the mirror of more aggressive decongestion and high diuretic administration, reflecting a temporal reduction of renal perfusion pressure and kidney overload. Persistent AKI is likely due to effective hemodynamic impairment, primitive renal injury, and more intense neurohormonal activation, but this relation is not persuasive enough and deserves specific analysis. A systematic approach able to recognize the two typologies of AKI (i.e., transient versus persistent and primary versus secondary) should lead to a more appropriate diuretic administration. Understanding the exact hemodynamic mechanism of congestion, its nature and severity is a further target to detect the correct diuretic dosage.

## Loop diuretics and outcome

The administration of intravenous loop diuretics to patients with heart failure (HF) and congestion results typically in a prompt diuretic effect. In most patients, the increased diuresis is accompanied by a decrease in LV ventricular filling pressures and improvement of symptoms by reducing pulmonary capillary wedge pressure and intra-alveolar edema [25]. When used in combination with vasodilators, loop diuretics reduce ventricular remodeling and mitral regurgitation, resulting in increased cardiac output. In spite of these hemodynamic effects, no long-lasting benefits were demonstrated with this approach [26]. In population studies, after adjustments for possible confounders, the highest diuretic quartile of loop diuretic dosing remained a significant predictor of mortality [26]. An increased risk for in-hospital mortality and renal failure has been consistently associated with higher doses of intravenous loop diuretics compared with lower doses [27, 28]. The Acute Decompensated Heart Failure Registry (ADHERE) analysis confirmed that patients receiving the lower doses had a lower risk of in-hospital mortality, intensive care unit stay, prolonged hospitalization, or adverse renal effects [29]. A meta-analysis by Abdel-Qadir et al. including more than 4000 patients has demonstrated a clear relation among discharge diuretic dosage and recurrent events over a mean 3-year follow-up period and shown doubled mortality in the highest quartile compared with the lowest [30]. In accordance with this study, the ESCAPE Trial showed that patients submitted to higher daily dosage (300 mg/day) had an unfavorable laboratory pattern in terms of elevated natriuretic peptide levels, increased creatinine and hyponatremia, and worsened clinical outcomes. In the same analysis, the authors observed an inverse linear relation between diuretic dosage and adverse outcome and suggested the lowest dosage feasible to resolve congestive symptoms [7]. As mentioned above, these associations could all represent confounding by indication, meaning that the more severely ill require higher doses of diuretics, particularly when urine output does not respond, and hence could be at higher risk for adverse outcomes independent of the utilization of loop diuretics. As mentioned above, the DOSE trial, a prospective randomized double-blind controlled trial with a two-by-two factorial design that compared bolus versus continuous infusion and low-dose versus high-dose strategies in a large population, did not demonstrate any benefit (death or hospitalization) of one strategy with respect to the others: analysis regarding bolus versus continuous administration revealed a trend toward a higher rate of creatinine increase in the continuous arm, although this has not influenced outcome [21]. Finally, a recent Cochrane analysis showed that bolus infusion was related to both increased urine output and fewer adverse effects compared with continuous infusions; unfortunately, no data were reported on long-term mortality and post-discharge events [31]. Thus, it remains unclear whether high doses or continuous infusions of loop diuretics cause harm; but given all of the data to date, they are very unlikely to help patients. Taken together, these findings suggest that higher dosage administration of loop diuretics probably contributes to adverse events as well as serving as a proxy for more severe disease which itself confers a poor prognosis. This theory seems to be confirmed by a subanalysis of the DOSE trial showing that patients with higher furosemide dose experienced worse renal function, more advanced symptoms, and New York Heart Association class. Besides, the same authors demonstrated a close relation between higher diuretic dose and risk of rehospitalization [32]. Further studies are warranted to determine whether high-dose diuretics are responsible for worsening renal function and whether coexisting renal dysfunction could be a marker of more severe HF.

## Loop diuretics, decongestion, and acute kidney injury

Baseline renal function and the dynamic change in serum creatinine are related to short- and longer-term outcomes. A recent observational study showed that creatinine change during hospitalization was independently associated with a higher risk of 1-year mortality. However, in patients with normal or mildly impaired renal function, creatinine changes (ΔCr) (admission to 48–72 hours) were not significantly associated with mortality. Interestingly, in this subgroup of patients, a decrease in ΔCr was associated with worse outcomes. Perhaps a transient deterioration of renal function is required to relieve congestion and improve global symptom assessment. Thus, the intricate cardio-renal axis relation is much more confused by the diuretic administration that could potentially amplify deleterious effects at the kidney level. Studies in this field revealed contrasting results: some authors did not observe a poor prognosis during treatment in patients who experienced worsening renal function [33]. Others believe that impaired renal function during treatment is only the consequence of aggressive fluid loss and decongestion. Aggressive decongestion associated with plasma hemoconcentration is associated with significant mortality reduction at 6 months, although an increase in creatinine was observed [34, 35].

The only two studies evaluating congestion and renal function during different diuretic strategy administration are the DOSE trial and the Diur HF trial. In the DOSE registry, patients submitted to higher dose and continuous modality administration revealed an increase in creatinine, although they experienced improvement in their symptoms and significant decongestion signs compared with low-dose administration. However, this study did not clarify which treatment modality is better to improve outcomes and renal function and showed no significant difference in hard endpoints during the follow-up period. Therefore, the authors did not report interactions between patients’ baseline characteristics and the efficacy of either intervention. The average dose administered was approximately 100 mg every 12 hours, and the average infusion was around 10 mg/hour [21]. In clinical practice, particularly in patients with renal dysfunction and diuretic resistance, the more appropriate dose would be up to 200 mg every 12 hours. Finally, follow-up monitoring was stopped at 60 days, and no data regarding changes in renal function during post-discharge period are available.

Conversely, in the Diur HF Trial, mean diuretic regimen was higher with respect to the DOSE patients; therefore, continuous versus bolus administration was compared. An increased rate of adverse outcome in patients developing renal impairment during hospitalization was observed. This occurs despite a significant reduction of B-type natriuretic peptide (BNP) level and urine output increase found in the continuous arm [22]. Although these tests are excellent in aiding the clinical determination of AHF and providing prognosis, it has been shown that a reduction in BNP/N-terminal of the prohormone brain natriuretic peptide (BNP/NT-proBNP) is not necessarily associated with freedom from AKI or a reduction in the risk for readmission or death over the short term. By current analysis, it is not possible to define the better modality administration and dosing regimen: higher dose should be prospectively tested monitoring decongestion by weight loss, diuresis, and symptom score measurement together with in-hospital and post-discharge renal function determination.

## Pitfalls and potential strategies during treatment

**Fig. 2:**
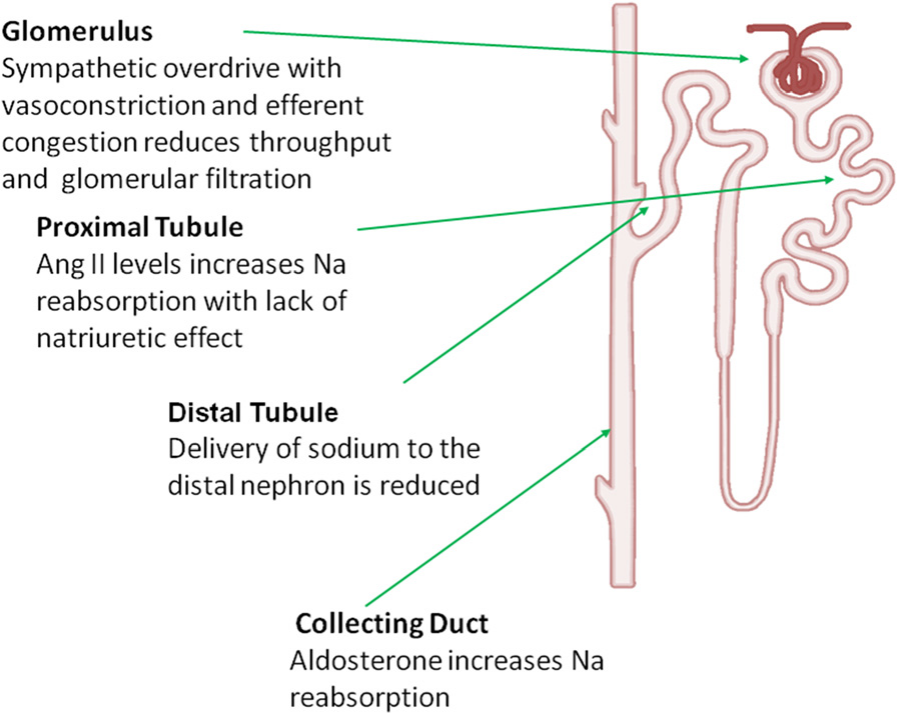
Effects of renin-angiotensin-aldosterone and sympathetic overdrive in different kidney sites

Treating and solving congestion are among the principal goals of AHF management. Persistence of congestion and poor response to diuretic treatment are associated with increased risk of mortality and threefold higher rate of re-hospitalization [36, 37]. Because higher-dose regimens have been related to adverse outcome, relevant questions remain about the optimal use of diuretics to obtain efficient decongestion. A post-hoc analysis from DOSE and Cardiorenal Rescue Study in Acute Decompensated (CARRESS) HF highlighted that higher levels of plasma renin activity were associated with lower blood pressure, azotemia, and hyponatremia in more advanced HF [38]. A report by Testani et al. showed that BUN/creatinine ratio but not creatinine increase alone was associated with early adverse events [39]. These studies suggest that neurohormonal activation and plasma volume contraction due to poor plasma refill are major determinants of short-term complications, including hypotension, AKI, and hyponatremia [40] (Fig. 2).

To avoid these pitfalls, many strategies have been proposed. The so-called breaking phenomena of loop diuretic resistance could in theory be overcome by continuous infusion. Sequential nephron blockade includes the contemporary use of loop diuretics together with thiazides and aldosterone antagonists in order to obtain a stepwise impairment of sodium reabsorption at multiple points along the nephron. This strategy is prone to increased risks of hypoperfusion, hypokalemia, and hyponatremia [41]. Lastly, ultrafiltration of isotonic fluid could in theory remove fluid at a rate to match plasma refill without causing volume contraction or activation of neurohormonal systems [42]. This method may be beneficial in congestive patients who are predicted to become diuretic-resistant. Another optional strategy during early phases of AHF management is the non-invasive ventilation (NIV) therapy. Although the potential beneficial role of the current treatment is still under debate, it is accepted for patients with low systolic pressure values to improve respiratory gas exchanges. Prior studies have been limited to patients with cardiogenic shock and pulmonary edema and have provided conflicting results: data from the ADHERE registry documented a reduced mortality rate with respect to patients with pulmonary edema submitted to endotracheal intubation together with reduced length of hospital stay [43]. More recently, a cross-sectional multicenter study confirmed previous findings underlying some difference in the use and application of modalities [44]. Finally, the NIV treatment seems to have beneficial effects, reducing the need for vasoactive and inotropic therapies. All of these data suggest that NIV should be a reasonable treatment option in some specific AHF pictures characterized by systemic hypoperfusion and low oxygenation status [45]. NIV could potentially favor the reduction of high dosage loop diuretic administration, avoiding further decrease in blood pressure, although specific studies appear mandatory to clarify the specific classes which deserve this treatment.

**Fig. 3:**
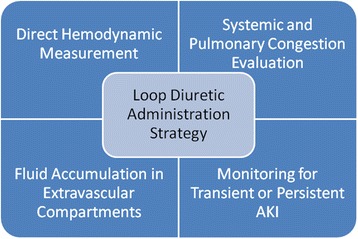
Strategy for loop diuretic therapy optimization looking for renal dysfunction fluid accumulation and hemodynamic status. *AKI* acute kidney injury

In the setting of neurohormonal activation and adequate perfusion, nesiritide could play an adjunctive role but does not have a broadly applied benefit in AHF [46]. It is possible that oral tolvaptan used in those with high arginine vasopressin (copeptin) levels even in the absence of hyponatremia could be beneficial in prompting an aquaresis before congestion worsens [47]. Both of these approaches would call for a personalized approach which is based on hemodynamic and biomarker profiles and which has not been undertaken in clinical trials to date. Additionally, methods to anticipate diuretic resistance before it occurs are needed to avoid excessive loop diuretic use with either sequential pharmacologic nephron blockade or ultrafiltration [48, 49]. Overall, a direct measurement of hemodynamic changes in arterial and venous districts to better understand the primary dysfunction and the venous or pulmonary congestion should be applied by central venous pressure estimation and LV filling and pulmonary pressure exertion. Congestion status and intra- or extravascular fluid accumulation are other important items deserving careful monitoring. This is actually possible by the routine use of bioimpedance, evaluation of vena cava collapse, and lung ultrasound [10] (Fig. 3). Such tools will likely include advanced renal physiologic imaging, advanced assessments of systemic and hemodynamics in the intra-abdominal arteries and veins, blood and urine biomarkers of kidney tubular function and injury, and lastly assessment of systemic neurohormonal activation profiles (renin-angiotensin, arginine vasopressin, sympathetic nervous system, endothelin, and others).

## Conclusions

Pulmonary and systemic congestion are inextricably linked to renal function. Use of loop diuretics is clearly playing a role in decongestion but at the same time is probably precipitating AKI and type 1 cardiorenal syndromes in patients at risk for worsened renal function and poor outcomes. Future research using advanced tools and personalized approaches for optimal outcomes is needed. Broad applications of high-dose or continuous infusion of loop diuretics or adjunctive use of inotropic and new vasoactive therapies have not been associated with improved outcomes. We propose an algorithm including a metric measurement of decongestion with concomitant evaluation of renin-angiotensin-aldosterone system activity and renal dysfunction mechanisms. This approach would allow a better AHF treatment by actualizing the integrated actors of the cardio-renal axis.

## Abbreviations

ADHERE: Acute Decompensated Heart Failure Registry
AHF: Acute heart failure
AKI: Acute kidney injury
BNP: B-type natriuretic peptide
BUN: Blood urea nitrogen
ΔCr: Creatinine changes
DOSE: Diuretic Optimization Strategies Evaluation
ESCAPE: Evaluation Study of Congestive Heart Failure and Pulmonary Artery Catheterization Effectiveness
HF: Heart failure
LV: Left ventricular
NIV: Non-invasive ventilation
